# JEnsembl: a version-aware Java API to Ensembl data systems

**DOI:** 10.1093/bioinformatics/bts525

**Published:** 2012-09-03

**Authors:** Trevor Paterson, Andy Law

**Affiliations:** Division of Genetics and Genomics, The Roslin Institute and Royal (Dick) School of Veterinary Studies, University of Edinburgh, Easter Bush, Midlothian EH25 9RG, UK

## Abstract

**Motivation:** The Ensembl Project provides release-specific Perl APIs for efficient high-level programmatic access to data stored in various Ensembl database schema. Although Perl scripts are perfectly suited for processing large volumes of text-based data, Perl is not ideal for developing large-scale software applications nor embedding in graphical interfaces. The provision of a novel Java API would facilitate type-safe, modular, object-orientated development of new Bioinformatics tools with which to access, analyse and visualize Ensembl data.

**Results:** The JEnsembl API implementation provides basic data retrieval and manipulation functionality from the Core, Compara and Variation databases for all species in Ensembl and EnsemblGenomes and is a platform for the development of a richer API to Ensembl datasources. The JEnsembl architecture uses a text-based configuration module to provide evolving, versioned mappings from database schema to code objects. A single installation of the JEnsembl API can therefore simultaneously and transparently connect to current and previous database instances (such as those in the public archive) thus facilitating better analysis repeatability and allowing ‘through time’ comparative analyses to be performed.

**Availability:** Project development, released code libraries, Maven repository and documentation are hosted at SourceForge (http://jensembl.sourceforge.net).

**Contact:** jensembl-develop@lists.sf.net, andy.law@roslin.ed.ac.uk, trevor.paterson@roslin.ed.ac.uk

## 1 INTRODUCTION

The Ensembl Project provides a genome information system for the annotation, analysis and display of genome assembly data pertaining to vertebrates [ENSEMBL (http://www.ensembl.org)] and for other taxonomic groups [ENSEMBLGENOMES (http://www.ensemblgenomes.org)]. Together with core genomic annotations, the curated resources now include comparative genomic, variation, functional genomic and regulatory data stored in separate but linked database schemas (Flicek *et al.*, 2010).

Access to data in Ensembl databases is freely provided through Ensembl’s interactive web browser, the BioMart data mining tool (http://www.ensembl.org/biomart/martview) and web services (http://www.biomart.org/martservice.html), publicly exposed MySQL databases (ensembldb.ensembl.org:5306; mysql.ebi.ac.uk:4157; ensembldb.ensembl.org:3306) and programmatically through Perl API modules (http://www.ensembl.org/info/data/api.html) (Stabenau *et al.*, 2004). The Perl API is ideally suited to the processing of large volumes of text-based data and as such is used for the majority of the Ensembl systems’ internal workflows. However, Perl is not an ideal language for embedding in graphical interfaces.

In contrast, Java provides a robust, object-oriented programming environment and is a preferable language for implementing large-scale projects, with the benefits of compile time type checking, enforced interfaces, the potential to separate interface from implementation (allowing for transparent alternative implementations), multi-threading, better support for graphical user interfaces and support for garbage collection of circularly referenced objects. Java, like Perl, also benefits from a vast resource of freely available diverse code libraries and development frameworks and tools, including open source projects in the Bioinformatics domain.

Previously, Ensembl provided the ENSJ library, a Java API for data access in Java or Jython (Stabenau *et al.*, 2004). Like the Perl API, ENSJ intimately embedded data access code (i.e. the actual SQL statements that access the Ensembl MySQL databases) within the body of code representing the genetic objects. As a consequence, a new API release had to be generated for each database schema version (Ensembl release) configured to connect and retrieve data from its cognate schema, with no backwards compatibility guaranteed. As with the Perl API, the dispersed nature of the embedded SQL statements meant that changes required to keep pace with each database release were spread across multiple files, which was an inefficient process. In 2006, ENSJ was discontinued when the Ensembl team elected to focus their finite resources on the maintenance of the Perl API code alone. Since then, despite the growing number of bioinformatic tools being developed in Java, there has been no Java Ensembl API available.

Other parties have, however, developed several partial APIs to Ensembl in a number of alternative programming languages, particularly to support bulk data download. Typically, these APIs do not directly address the issue of schema versioning, and many are not actively maintained. Two of the most widely used such APIs are the Bioconductor R interface to BioMart, ‘biomaRt’ (http://www.bioconductor.org/packages/release/bioc/html/biomaRt.html), although this is a biomart API rather than an Ensembl API *per se*, and the BioGem plug-in ‘ruby-ensembl-api’ (http://bioruby-annex.rubyforge.org/) which uses Active Records to abstract over the Ensembl Core Schema. Although the ActiveRecord design allows the API code to remain ‘in sync’ with the database schema automatically, no higher level data model is generated and scripts that run against a particular release of the Ensembl databases will not run against other releases if the names of tables or columns have been changed. Several Python-based APIs that have been made available have not evolved with schema changes and provide limited data models [e.g. PyCogent (http://pycogent.sourceforge.net) (Knight *et al.*, 2007), PyGr (http://code.google.com/p/pygr/wiki/PygrOnEnsembl), cache-ensembl (http://pypi.python.org/pypi/cache_ensembl)].

A new, easily maintainable Java-based API to the Ensembl system would be a timely and highly effective addition to the bioinformatics toolbox. Such an API would allow integration between graphical user interfaces and Ensembl datasources and between other bioinformatic resources and libraries implemented in Java [for example, the BioJava (http://www.biojava.org) framework; Holland *et al.*, 2008].

A full Java API to the Ensembl system would replicate all the data access functionality of the Perl API Core, Compara, FuncGen and Variation modules and would:

1. Connect to and extract data from the current release version of Ensembl.
2. Access all instances of Ensembl data systems including single-species databases at Ensembl and EnsemblGenomes and the multi-species databases (bacterial collections) at EnsemblGenomes.
3. Access data from all database types: Core, FuncGen, Variation, Compara, etc.
4. Emit software objects corresponding to the major object types within Ensembl including Sequence Regions, Markers, Alleles, Genes, Exons, Transcripts, CoordinateSystems and AnnotationFeatures of numerous kinds.
5. Map between appropriate CoordinateSystem levels for a given genome (thus allowing actual DNA sequence data to be retrieved for features annotated at higher levels, e.g. genes on chromosomes).
6. Provide an architecture for updating the API connectivity and functionality as new versions of Ensembl are released, while maintaining backwards compatibility with earlier releases (improve on the Perl requirement for version-specific API releases).
7. Be compatible with (and build upon) existing open source Java libraries for bioinformatics where relevant (e.g. BioJava 3.0).

We report here the implementation of an extendable Ensembl Java API that demonstrates the tractability of the objectives above; specifically it provides access to all versions of databases currently published at Ensembl and EnsemblGenomes. It implements core functionality for the retrieval of chromosome, gene, transcript, exon, protein data, etc. from ‘Core’ databases; maps locations between CoordinateSystems and maps transparently between database versions where changes in the schema necessitate different SQL statements to extract the same information. We have also implemented retrieval of SNP variation information from the ‘Variation’ databases and comparative homology information from the ‘Compara’ databases.

We demonstrate the potential utility of our Ensembl Java API by incorporating the JEnsembl libraries in a plug-in created for the Savant Genome Browser (http://www.savantbrowser.com/) (Fiume *et al.*, 2010), and in our Genetic Map Drawing application ‘ArkMAP’ (http://www.thearkdb.org/arkdb/download.jsp). These plug-ins demonstrate how third-party developers can use JEnsembl to access data from Ensembl datasources, allowing the graphical display and alignment of chromosomal sequences, variations and exceptions, gene annotations and gene homologies.

## 2 IMPLEMENTATION

**Fig. 1.**
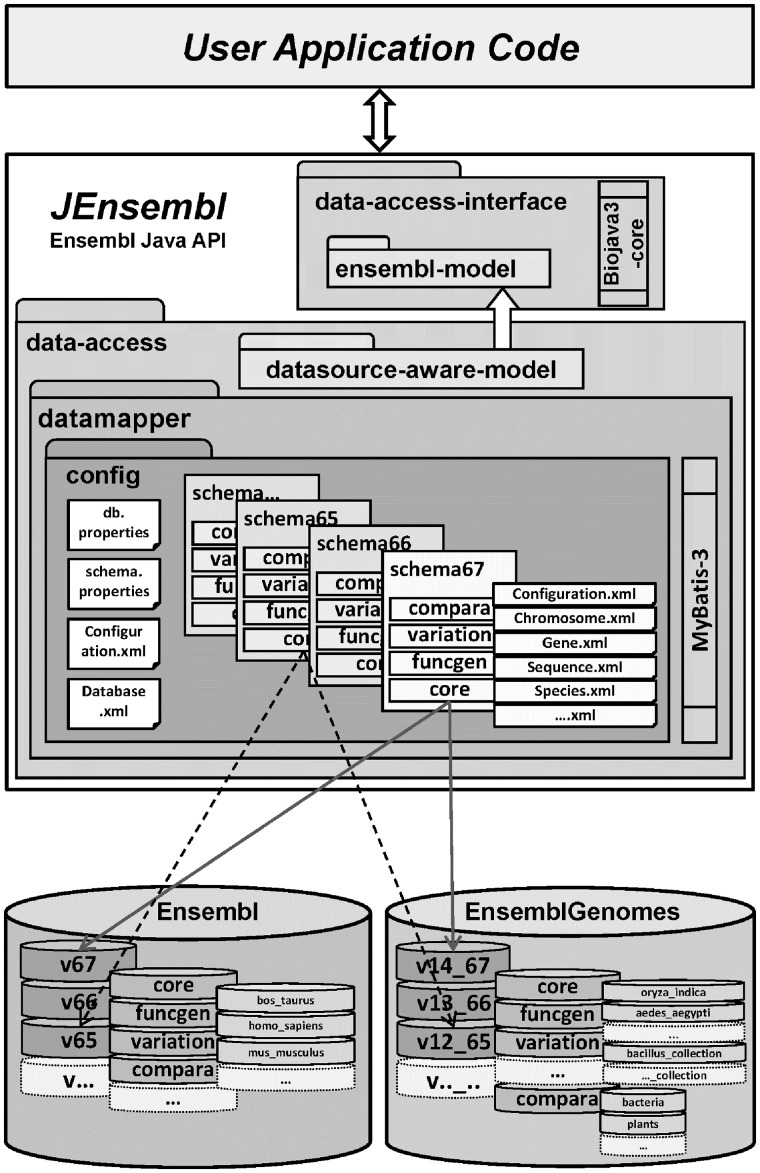
JEnsembl architecture. Schematic diagram of the modular JEnsembl architecture, where schema-versioned MyBatis configurations in the ensembl-config module are mapped to DatasourceAware objects using the MyBatis data mapping framework. Connection to external Ensembl datasources is via the MySQL JDBC connector

JEnsembl is implemented in Java version 1.6 following a modular design pattern using Maven software management. Project development is hosted on SourceForge where code is available from the subversion repository (http://jensembl.sourceforge.net/; https://sourceforge.net/projects/jensembl/). The architecture of the project is shown schematically in Figure 1. Each of the separate interdependent modules of the API is built as a Maven artifact allowing for public distribution via Maven repositories. Alternatively, the Jar artifacts can be used as standard Java libraries outwith a Maven build environment. Each module is coded against full JUnit tests, with an additional module providing demonstration code and functional tests for data retrieval by the API from remote datasources. Current release versions of the libraries are available on the project website and Maven repository.

**Fig. 2.**
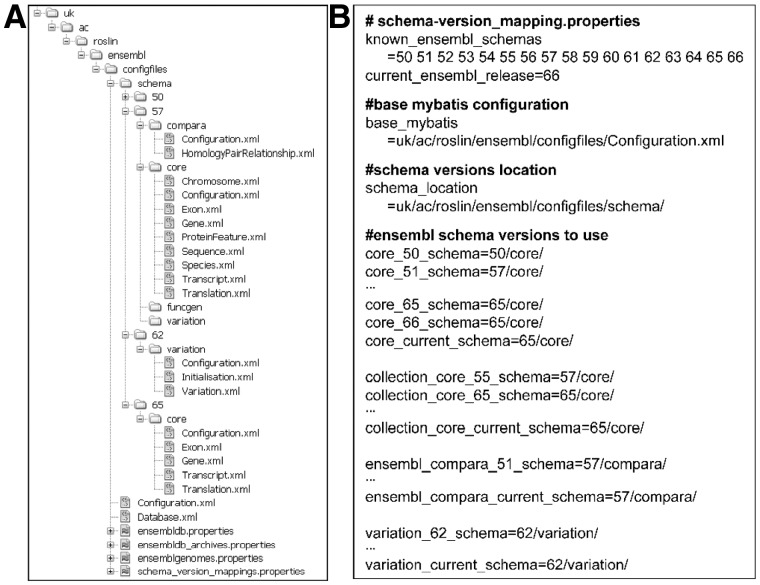
Data mapping between database releases and schema versions. (**A**) The configuration file hierarchy in the *ensembl-config* module. The *ensembldb*, *ensembldb-archives* and *ensemblgenomes* properties files hold JDBC connection parameters, while *schema_version_mappings* specifies which MyBatis configurations are to be used for each Ensembl release version. The base *Configuration.xml* and *Database.xml* files configure connection at the datasource level, while release-specific MyBatis mappings are held in database type-specific directories: *schema/XX/compara*, *core*, *funcgen* and *variation*; rules specified in a *Configuration.xml* file in each directory allows a release configuration to use mapping files from different directories. (**B**) Abridged listing of *schema_version_mappings* properties, showing how the appropriate mappings of database type and version to MyBatis configuration directories are specified. Core and Compara mappings were developed for release 57 and are backwards compatible to release 51. Variation mappings were introduced from version 62 and Core mapping rules updated at release 65

The modular design of the JEnsembl artifacts is described more fully in the online documentation. In brief, the JEnsembl API defines Java objects corresponding to the various genetic objects described in the Ensembl datasources (i.e. Chromosomes, DNASequences, Features, Species, Genes, etc.). These data objects are created and populated through the data access layer (see Fig. 1) using MyBatis (http://www.mybatis.org/) as the RDBMS-to-Java object-mapping tool. A fundamental goal of the project design was to separate schema version-specific database query code from the data model; this is achieved by partitioning the SQL code and MyBatis data mapping rules into a hierarchy of XML configuration files in the configuration module (see Fig. 2). Configurations in the *schema.properties* file automate which mapping rules are used for each Ensembl release-version, allowing the data access code seamlessly to maintain correct data mappings as the Ensembl data schema evolves, while retaining backwards compatibility with earlier schema.

To connect to a datasource (e.g. Ensembl), a *DBRegistry* object is instantiated by injecting either a default *RegistryConfiguration* object read from the current *ensembl-config* module or a *RegistryConfiguration* generated from locally supplied properties. The *RegistryConfiguration* defines the set of MyBatis configuration files to read for each version of the database/schema identified within the installation. Upon DBRegistry initialization, the names of available databases at the configured datasource are parsed using the Ensembl naming conventions and meta-data tables to identify database-type, species, assembly and schema release versions. The DBRegistry object can then be queried for lists of known databases or species, or can return objects extracted from current or specific releases of named species databases.

JEnsembl represents each of the database schema with a hierarchy of subclasses of the Database class, (CoreDatabase, ComparisonDatabase, VariationDatabase, etc.). A correctly typed instance of a Database class is constructed by the Registry for each species/version/schema database, with each Database object creating its own instance of a MyBatis SqlSessionFactory, configured (via the Configuration artifact) with the correct SQL mapping files for the appropriate schema type and version. Correctly configured data access is controlled by DAOFactory objects; an appropriate type of DAOFactory is created on demand for each Database instance and automatically configured to use the correct MyBatis mapping rules for its schema version. The DAOFactory provides the DAO access objects which perform data queries using MyBatis SqlSessions provided by their shared DAOFactory. These SQL queries typically return DatasourceAware objects; each DatasourceAware object holds a reference to its own DAOFactory, which is used to perform lazy loading of data fields and perform queries about further data relationships. Hence, all access to a particular database is effectively performed through a DAOFactory singleton (providing the opportunity for implementing data caching).

Databases with schema versions for which configuration details are not explicitly provided will not be made available by the Registry, thus avoiding incompatibility with unsupported older releases or newer releases that post-date the API code and that have not yet been mapped. New Ensembl releases requiring changes to SQL code are handled simply by defining a new mapping configuration. Where no changes are needed, existing configurations can be reused in a flexible and granular fashion—new mapping configurations can import existing elements and only need to replace the individual mapping files that cover the modified part of the schema. This architecture is illustrated in Figure 2.

The Ensembl datasources contain not only the actual DNA sequences of genome assemblies but also annotations of features on the assembly derived from Ensembl’s own pipeline analyses and external sources, together with derived relationships between these features. Core sequence and assembly information together with gene and transcription annotations are stored in a ‘Core’ schema, while the other (optional) data schema are used to hold further information about the better studied model species. Access to data in the other (non-Core) database schema is controlled through the Core DAO Factory, which, for example, can supply an instance of a DAOVariationFactory for the correct species/version Variation Database, with its own correctly configured SQLSessionFactory. This DAOVariationFactory supplies a DAOVariation object, which may be used to retrieve all the variations for a given chromosomal region. Comparative genomic data are stored somewhat differently in Ensembl, and a DAOComparaFactory accesses a single Compara database for each release of Ensembl, which holds the results of pair-wise inter-species comparisons (comprising both genomic alignments and gene family and homology data).

The EnsemblGenomes datasource uses the same (versioned) schema as Ensembl (which is now focused as a Vertebrate resource), but with species organized into five separate taxonomic groups, each with its own Compara database. Therefore, as with the Ensembl Perl API, JEnsembl can use the same API for data access from EnsemblGenomes with the added benefit of version aware configuration on the fly. However, EnsemblGenomes bacterial datasources differ significantly in being organized into multi-species databases according to phylogeny. Ensembl adapted their schema to handle multi-species resources, and the Perl API handles all schema identically (as potentially multi-species). In JEnsembl, multi-species resources are currently handled by implementing separate ‘multi-species’ interfaces in Database and Factory objects. Because the underlying schema is identical, the multi-species data access architecture could be used for accessing standard single-species datasources. However, currently we feel retaining the single-species database paradigm is simpler for the majority of users and allows for easier representation of a ‘species’ object, shared between database release versions.

In order to harness the comprehensive sequence manipulation features of BioJava libraries, we extended the BioJava 3.0 Core DNASequence object for the JEnsembl DNASequence object, providing an Ensembl SequenceReader that can lazy-load sequence on demand from the Ensembl datasource. This provides the JEnsembl Sequence objects with BioJava API behaviour, for example reading protein sequences from translated transcripts. Incorporation of third-party open source libraries not only obviates code duplication but also enables interoperability with a wider range of third-party software.

The JEnsembl release libraries were used to create a novel plug-in for the Savant Genome Browser (Fiume *et al.*, 2011). The plug-in source code and binary Jars for different versions of the browser are also available from the JEnsembl project site on SourceForge (http://jensembl.sourceforge.net/savant.html). Our ‘ArkMAP’ map drawing tool has recently been converted to retrieve chromosome gene annotation data directly from Ensembl datasources using the JEnsembl API instead of the BioMart web services, thus allowing ArkMAP to be ‘Version Aware’ for Ensembl data. JEnsembl-mediated access to Compara data allows the discovery and alignment of regions of conserved synteny between species and SNP marker mappings can be retrieved from Variation datasources.

## 3 RESULTS AND DISCUSSION

### 3.1 JEnsembl

The JEnsembl development code, Jar library releases (Maven artifacts) and documentation including JavaDocs are available on SourceForge (current release 1.12). Access to an Ensembl datasource is achieved by initializing a DBRegistry object either with one of the two configurations provided (ENSEMBLDB or ENSEMBLGENOMES) or with user-specified configuration properties that allow connection to alternate datasources using the Ensembl schema, for example Ensembl Archives or private, local data resources. Initialization of the Registry object sorts and registers the available databases at the selected datasource: their release number, schema type and species, determining which releases match the schema version mappings in the current JEnsembl Configuration module. Thereafter, data from any ‘known’ database type and version can be interrogated through the Registry. In the absence of specified type or version number, a query retrieves data by default from the most recent configured (i.e*.* ‘known’) version of the appropriate database type. In addition to providing public access to the databases, the Registry provides public access to Species objects by name or alias, suitable for more high-level usage. Species can then be queried for information about genes, sequences, etc. without any knowledge of the Ensembl data structure.

**Fig. 3.**
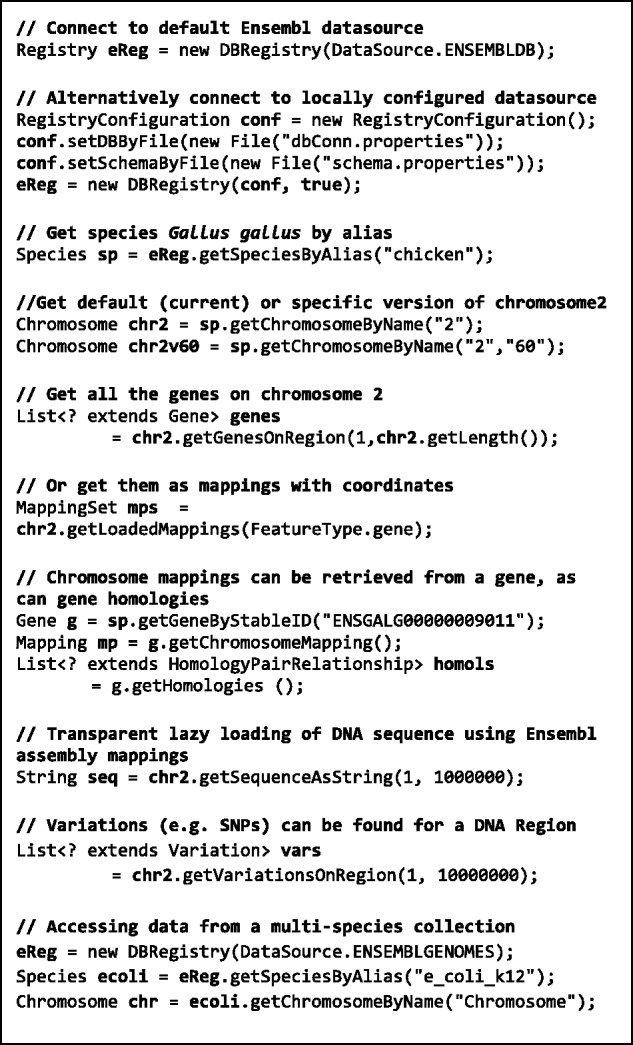
Example usage of JEnsembl Java API (v1.12). The Species ‘ecoli’ retrieved in the final code block is actually a CollectionSpecies because it is retrieved from the ‘escherichia_shigella_collection_core’ databases. CollectionSpecies are slightly less reliable access points than normal Species as there is no guarantee of stable species, strain names and aliases between releases

**Fig. 4.**
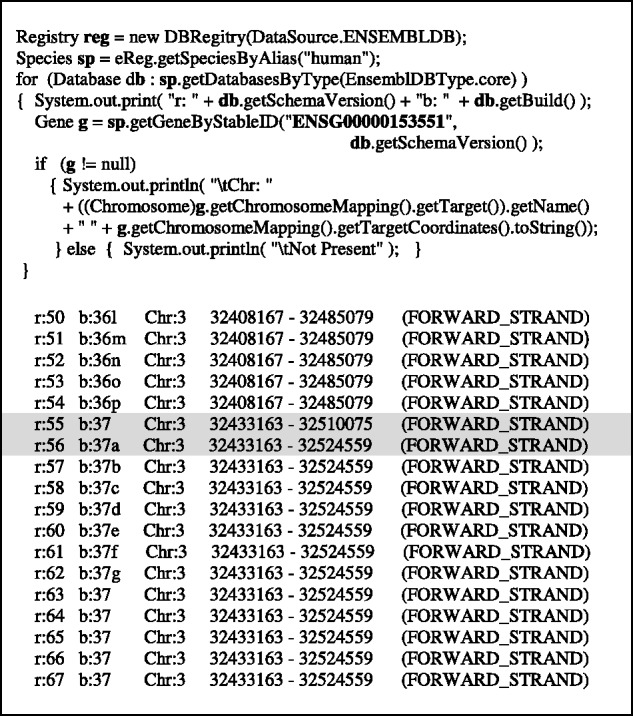
Code illustrating JEnsembl API retrieving chromosomal coordinates for a human gene (Ensembl ID ENSG00000153551) for 18 different Ensembl Releases currently available at the ENSEMBLDB datasource (i.e. MySQL databases at ensembldb.ensembl.org:5306). The results reflect different coordinates of this gene in assembly builds 36 and 37. The increase in apparent gene size between release 55 and 56 (highlighted) is due to the addition of further transcripts to the gene model

Figure 3 demonstrates example code usage, starting with Registry initialization and retrieval of a Species object, which is then used to access data from specified release versions of the Ensembl datasource. Thus, the current or any earlier release version of chicken chromosome 2 (together with all of it annotations) can be retrieved (e.g. release ‘60’ in Fig. 3). This allows reproducible access to the correct version of data used by historical analyses and allows comparison of different versions of the data using a single API code installation. This is illustrated by the single code snippet shown in Figure 4 where data pertaining to a single human gene can be retrieved from the current and previous 17 human core database releases available at the Ensembl datasource (and for which the JEnsembl API has configured schema mappings). This allows, for example, the location of the gene to be compared over time, between Ensembl releases, assembly builds, patches and changes to the gene model and permits many other ‘through-time’ analyses of genome assemblies to be contemplated. The retrieval of similar multi-release data using Perl would require multiple, separate, release-specific versions of the Ensembl Perl API to be installed and involve complex library path manipulations.

A central concept within the JEnsembl model is a ‘Mapping’: between source and target ‘MappableObjects’, with source and target coordinates (start, stop, strand). Mappings allow any of the Ensembl annotation types to be located on other types (e.g. genes, exons, variations on chromosomes). When genes are retrieved from a chromosome, the query returns a set of genes, each with its own mapping to the chromosome, while the chromosome is returned with an ordered set of the (inverse) mappings of genes on the chromosome.

Retrieval of data across the database schema types is achieved transparently, by loading appropriate DAOFactories, and using appropriate identifiers shared across the schema. For example, querying a gene for homologues uses the gene’s stable Ensembl identifier to query the Compara schema, and the target ‘hits’ retrieved contain enough information to convert them to Core schema objects if required (target stable id, chromosome name and coordinates, and target species name). Similarly, queries to retrieve variants from the Variation database are parameterized with the Core sequence identifier and desired range coordinate and return the properties and mapping coordinates of variants within this range.

The Ensembl pipeline typically annotates features at the highest ‘level’ of coordinate systems used in production of the genome assembly. JEnsembl transparently integrates the varying levels of coordinate systems (chromosome, supercontig, contig, clone, etc.) down to the lowest ‘sequence’ level coordinate system. This is achieved using a hierarchy of interfaces: DNASequence, AssembledDNASequence and Chromosome. AssembledDNASequences contain an assembly of DNASequences (which may themselves be AssembledDNASequences) at a given coordinate system level. Hence, the actual DNA sequence for a given chromosome is returned by lazy loading the assembly mappings and underlying sequence level objects to retrieve the range of actual sequences required. The JEnsembl DNASequence classes are built upon BioJava3 DNASequence and extend the ProxySequenceReader interface to load, read and manipulate sequences.

Throughout the JEnsembl development process, Ensembl has continued to release successive versions of its datasets, with an evolving data schema. This evolving schema has afforded a challenging opportunity to demonstrate the effectiveness of JEnsembl’s transparent version configuration strategy. For example, a major change was introduced to the Core schema at version 51 to allow multiple species to be held within a single database, with separate coordinate systems being held for each species. Our code must therefore execute different SQL queries when retrieving coordinate system information from database instances before or after this release. Similarly, the merging of separate stable_id tables with the gene, exon, transcript and translation tables in Ensembl release 65 requires different SQL queries to be run post and prior this release. These schema migrations are specified in a hierarchy of MyBatis XML configurations and a properties file specifying which MyBatis configurations should be used for each schema release (see Fig. 2). The configuration occurs seamlessly and silently and requires no user intervention.

The JEnsembl development site details many more example code files that may be downloaded, and which demonstrate data access using all of the currently implemented aspects of the API. These files (found in the Ensembl Test artifact) include the data access routines used in the Savant and ArkMAP examples below.

### 3.2 Savant Plug-In

**Fig. 5.**
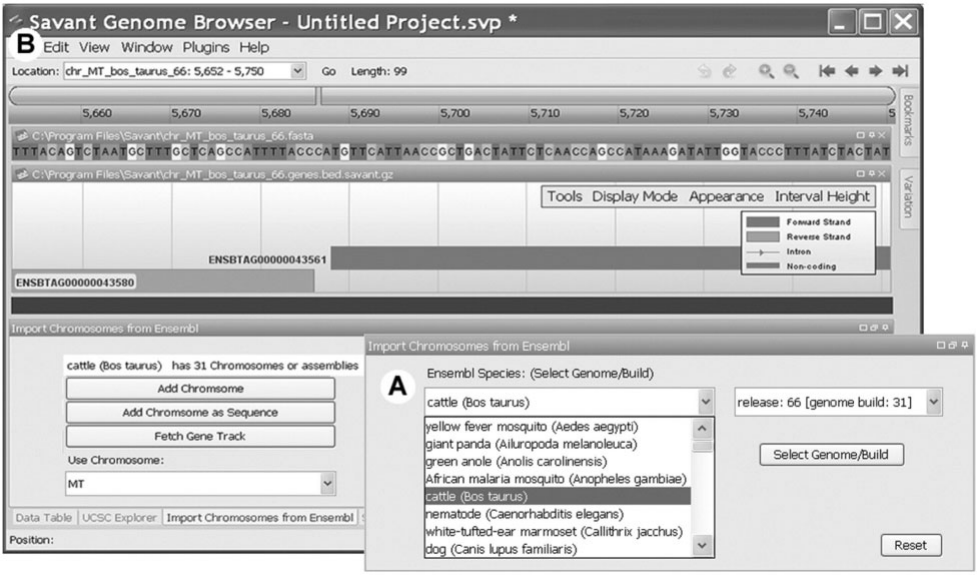
JEnsembl plug-in for Savant genome browser. (**A**) The user selects the desired species and release version from those available at the selected datasource (Ensembl, EnsemblGenomes or EnsemblGenomes-Bacterial). (**B**) A single chromosome/assembly is selected from those available for the chosen species/release. The chromosome is imported either as a simple coordinate skeleton or with the associated colour-coded genomic sequence. Currently, the only feature annotation that can be imported from the datasource is the gene track, which Savant shows aligned with the DNA Sequence

To demonstrate the potential utility of JEnsembl to third-party developers, we have implemented a Java plug-in Jar for the Savant Genome. The plug-in allows a Savant user to browse all of the available species and versions available at Ensembl and EnsemblGenomes, and to load chromosome assemblies for display in Savant. These can then be decorated with the gene annotations for that chromosome build (Fig. 5).

As in our code examples (Fig. 3), the plug-in creates a DBRegistry object and presents available databases (and subsequently chromosomes) to the user as drop down selection lists. The gene annotation data for the selected genome are retrieved from gene mappings (for example code, see Fig. 3) and then passed to the Savant application.

It should be noted that limitations in the Savant API architecture, whereby data must be passed in as a single BED file, preclude some of the capabilities of the JEnsembl code which has been designed to load sequence details in a ‘lazy’ fashion, i.e. only when needed. For this reason, importing of actual DNA sequence data together with the chromosome coordinates is provided as an optional step and should be avoided for large chromosomes.

### 3.3 ArkMAP

**Fig. 6.**
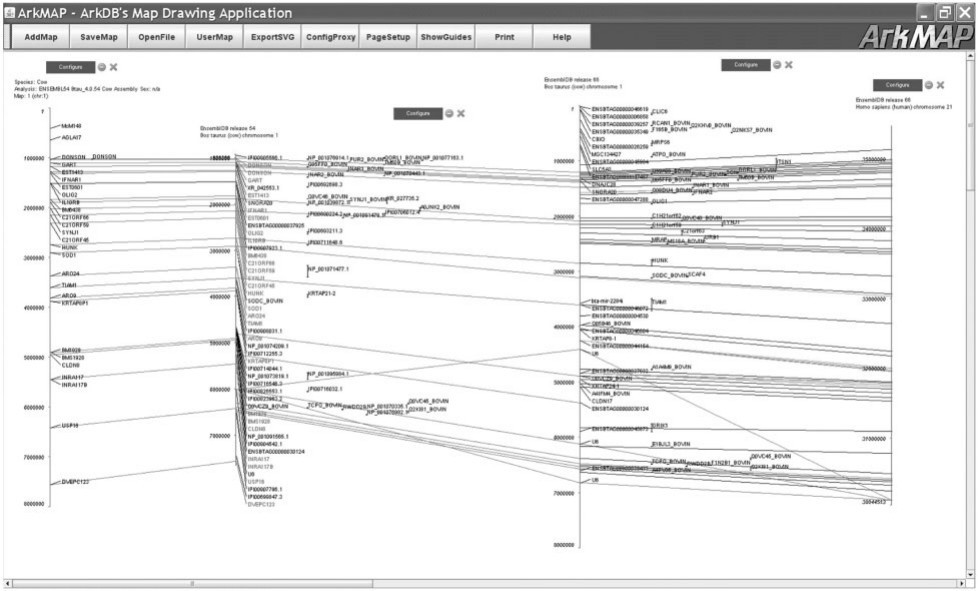
The ArkMAP application uses JEnsembl for retrieving maps and homologies from Ensembl datasources. ArkMAP can be used to draw genetic maps loaded from ArkDB, Ensembl or local datasources. Here the first 8 Mb of a bovine ePCR map has been loaded from ArkDB, where Ark Markers have been mapped on the Btau4 assembly. The JEnsembl API was then used to retrieve and align the cognate gene-annotated chromosome 1 assembly from Ensembl release 54. JEnsembl was then used to retrieve a more recent (release 66) gene annotated assembly which is aligned to the old assembly. Finally, JEnsembl was used to search for human gene homologies with the bovine genes in this region, and the region of conserved synteny on human chromosome 21 aligned with the bovine chromosome (with colour-coded homology relationships)

ArkMAP is a desktop Java application provided by ArkDB for drawing genetic maps (i.e. linkage maps, radiation-hybrid maps, cytogenetic maps, physical maps). It can download and align mapping data from ArkDB web services and from Ensembl datasources. It has recently been refactored to use the JEnsembl API to retrieve mapping data from JEnsembl. Previously, Ensembl assembly data (e.g. gene location annotations) were retrieved using BioMart web services, which restricted ArkMAP to accessing data in the current Ensembl release (held in BioMart). However, by using the JEnsembl API for data access, ArkMAP becomes release-version aware and data can now be selected for any available Ensembl release. This is important because it allows work performed using previous assemblies to be compared with the current genome assembly. This is illustrated in Figure 6: an ArkDB map created using the bovine assembly data of Ensembl release 54 can be aligned with gene annotation data from the appropriate Ensembl release, which can in turn be aligned with the most recent assembly release. The JEnsembl API allows additional data exploration: for example the discovery of gene homologies and the identification and alignment of regions of conserved synteny between species (as shown in Fig. 6) or the retrieval of the coordinates of SNP Markers (e.g. dbSNP markers).

### 3.4 Scripting with the JEnsembl API

The comprehensive Ensembl Perl API is widely used for data access and manipulation by bioinformaticians and Perl is an ideal scripting language for bulk sequence manipulation. However, as further functionality is implemented in JEnsembl, there is greater potential for using Java scripts for data manipulation, a task aided by the use of powerful Java IDE tools such as Netbeans and Eclipse for writing code. Example data access scripts can be found in the Ensembl Test artifact described above and are available on the project website. Use of JEnsembl has both validated the API and driven implementation of new features as required. For example, retrieval of variation data from the Variation schema databases was introduced to support a script that outputs SNPs within a given proximity to an annotated gene, while the requirement for a mechanism to retrieve pseudoautosomal sequences was exposed by scripts which were failing to locate sequence features on the human Y chromosome.

## 4 CONCLUSIONS

The majority of bioinformatic processing of genome information has traditionally been performed using Perl scripting, and the Ensembl Perl API is a fundamental tool for bioinformatic analysis. However, Java developers of bioinformatics tools, particularly graphical display interfaces, have been restricted by the lack of a generic Java API for accessing Ensembl data. In its absence, they have been forced to develop *ad hoc* solutions and data models for importing and representing genome data from Ensembl either directly accessing the raw MySQL datasources (which have an extremely complex data model) or retrieving data from Web Service calls to Ensembl BioMart and converting the raw data to the user’s own genetic data model. All of these methods are fragile to a lesser or greater degree and thus represent ‘workarounds’ rather than ‘solutions’. The provision of this Java API to Ensembl thus represents a valuable new resource for the expanding Java bioinformatics community.

Our current release version of JEnsembl demonstrates how we believe certain key aspects of a Java API should be addressed, in particular schema versioning and interoperability with other available Java libraries. It provides the framework on which to build a fully functional, open source implementation of a Java Ensembl API equivalent in functionality to the Perl API maintained by the Ensembl team. The project is hosted on SourceForge where we hope it will develop as a collaborative project similar to the BioJava code base and as such we call for and welcome expressions of interest from other developers.
